# Language deficits in specific language impairment, attention deficit/hyperactivity disorder, and autism spectrum disorder: An analysis of polygenic risk

**DOI:** 10.1002/aur.2211

**Published:** 2019-10-02

**Authors:** Ron Nudel, Camilla A. J. Christiani, Jessica Ohland, Md Jamal Uddin, Nicoline Hemager, Ditte V. Ellersgaard, Katrine S. Spang, Birgitte K. Burton, Aja N. Greve, Ditte L. Gantriis, Jonas Bybjerg‐Grauholm, Jens Richardt M. Jepsen, Anne A. E. Thorup, Ole Mors, Merete Nordentoft, Thomas Werge

**Affiliations:** ^1^ Institute of Biological Psychiatry, Mental Health Centre Sct. Hans, Mental Health Services Copenhagen Roskilde Denmark; ^2^ iPSYCH, The Lundbeck Foundation Initiative for Integrative Psychiatric Research Aarhus Denmark; ^3^ Mental Health Centre Copenhagen, University of Copenhagen Hospital Copenhagen Denmark; ^4^ Section for Biostatistics, Department of Public Health University of Copenhagen Copenhagen Denmark; ^5^ Mental Health Centre for Child and Adolescent Psychiatry – Research Unit, Mental Health Services in the Capital Region of Denmark Copenhagen Denmark; ^6^ Psychosis Research Unit, Aarhus University Hospital Risskov Denmark; ^7^ Center for Neonatal Screening, Department for Congenital Disorders Statens Serum Institut Copenhagen Denmark; ^8^ Center for Neuropsychiatric Schizophrenia Research and Center for Clinical Intervention and Neuropsychiatric Schizophrenia Research, Mental Health Services in the Capital Region of Denmark Copenhagen Denmark; ^9^ Department of Clinical Medicine Faculty of Health and Medical Sciences, University of Copenhagen Copenhagen Denmark

**Keywords:** polygenic risk score, specific language impairment, autism spectrum disorder, attention deficit hyperactivity disorder, genome‐wide association study

## Abstract

Language is one of the cognitive domains often impaired across many neurodevelopmental disorders. While for some disorders the linguistic deficit is the primary impairment (e.g., specific language impairment, SLI), for others it may accompany broader behavioral problems (e.g., autism). The precise nature of this phenotypic overlap has been the subject of debate. Moreover, several studies have found genetic overlaps across neurodevelopmental disorders. This raises the question of whether these genetic overlaps may correlate with phenotypic overlaps and, if so, in what manner. Here, we apply a genome‐wide approach to the study of the linguistic deficit in SLI, autism spectrum disorder (ASD), and attention deficit/hyperactivity disorder (ADHD). Using a discovery genome‐wide association study of SLI, we generate polygenic risk scores (PRS) in an independent sample which includes children with language impairment, SLI, ASD or ADHD and age‐matched controls and perform regression analyses across groups. The SLI‐trained PRS significantly predicted risk in the SLI case–control group (adjusted *R* ^2^ = 6.24%; *P* = 0.024) but not in the ASD or ADHD case‐control groups (adjusted *R* ^2^ = 0.0004%, 0.01%; *P* = 0.984, 0.889, respectively) nor for height, used as a negative control (*R* ^2^ = 0.2%; *P* = 0.452). Additionally, there was a significant difference in the normalized PRS between children with SLI and children with ASD (common language effect size = 0.66; *P* = 0.044). Our study suggests no additive common‐variant genetic overlap between SLI and ASD and ADHD. This is discussed in the context of phenotypic studies of SLI and related disorders. ***Autism Res** 2020, 13: 369–381*. © 2019 The Authors. *Autism Research published by International Society for Autism Research* published by Wiley Periodicals, Inc.

**Lay Summary:**

Language deficits are characteristic of specific language impairment (SLI), but may also be found in other neurodevelopmental disorders, such as autism spectrum disorder (ASD) and attention deficit/hyperactivity disorder (ADHD). Many studies examined the overlaps and differences across the language deficits in these disorders, but few studies have examined the genetic aspect thereof. In this study, we use a genome‐wide approach to evaluate whether common genetic variants increasing risk of SLI may also be associated with ASD and ADHD in the same manner. Our results suggest that this is not the case, and we discuss this finding in the context of theories concerning the etiologies of these disorders.

## Introduction

Specific language impairment (SLI) is a neurodevelopmental disorder characterized as a deficit in the development of language in an otherwise typically developing child [Bishop, 2006], with a prevalence of about 7% in kindergarten children [Norbury et al., 2016; Tomblin et al., 1997]. SLI has been shown to have a strong genetic component [Stromswold, 1998, 2001], and several studies have investigated its underlying genetic etiology using linkage [Bartlett et al., 2002; Falcaro et al., 2008; The SLI Consortium, 2002, 2004] as well as targeted association and genome‐wide association study (GWAS) designs [Newbury et al., 2009; Nudel, Simpson, Baird, O'Hare, Conti‐Ramsden, Bolton, Hennessy, Monaco, et al., 2014b; Nudel, Simpson, Baird, O'Hare, Conti‐Ramsden, Bolton, Hennessy, Ring, et al., 2014a; Vernes et al., 2008]. Other studies examined language traits and language deficits in population samples [Eicher et al., 2013; Luciano et al., 2013]. Exome sequencing studies of SLI have also been performed [Chen et al., 2017; Villanueva et al., 2015]. Combined, these efforts have resulted in several genes being identified as candidate genes for SLI or language traits, among them: *CNTNAP2* [Vernes et al., 2008], *ATP2C2* [Newbury et al., 2009], *CMIP* [Newbury et al., 2009], *NOP9* [Nudel, Simpson, Baird, O'Hare, Conti‐Ramsden, Bolton, Hennessy, Ring, et al., 2014a], *NFXL1* [Villanueva et al., 2015], *ABCC13* [Luciano et al., 2013], *ZNF385D* [Eicher et al., 2013], and several HLA genes [Nudel, Simpson, Baird, O'Hare, Conti‐Ramsden, Bolton, Hennessy, Monaco, et al., 2014b].

Interestingly, some SLI candidate genes have also been implicated in other neurodevelopmental disorders. For example, *CNTNAP2* has been highlighted in studies of autism spectrum disorder (ASD) [Alarcon et al., 2008] and attention deficit/hyperactivity disorder (ADHD) [Elia et al., 2009], and rare variants in *CNTNAP2* were found in children with childhood apraxia of speech [Worthey et al., 2013]; *CMIP* has been implicated in ASD [Van der Aa et al., 2012]; *ATP2C2* has been implicated in ADHD [Lesch et al., 2008]. HLA genes have been implicated in ASD and ADHD as well [Lee et al., 2006; Nudel et al., 2019; Odell, Warren, Warren, Burger, & Maciulis, 1997; Torres, Maciulis, Stubbs, Cutler, & Odell, 2002; Torres et al., 2006; Wang et al., 2008; Warren et al., 1996]. In addition to the genetic overlaps between ASD, ADHD, and SLI, it is known that language and communication may be impaired in ASD [Dover & Le Couteur, 2007] and ADHD [Baird, Stevenson, & Williams, 2000]. However, the exact nature of the language deficit may differ across disorders. Specifically, the relationship between SLI and ASD has been debated in the literature, with some evidence for shared linguistic deficits [Kjelgaard & Tager‐Flusberg, 2001] and some evidence for different linguistic profiles of children with SLI and children with ASD [Bishop, 2003a; Whitehouse, Barry, & Bishop, 2007; Williams, Payne, & Marshall, 2013]. Moreover, mostly due to clinical considerations, the label “specific language impairment,” which traditionally required some discrepancy between linguistic skills and nonlinguistic cognitive skills, e.g., verbal and nonverbal intelligence, and a lack of any other potential explanatory neurodevelopmental impairment, has recently been revised to allow the presence of some biological risk factors and the co‐occurrence of several other neurodevelopmental disorders, and to not require a discrepancy between verbal and nonverbal ability [Bishop, Snowling, Thompson, & Greenhalgh, 2017]. The new label adopted following this revision is “developmental language disorder” (DLD). Nonetheless, ASD remained an exclusion criterion for DLD [Bishop et al., 2017] due to its genetic etiology and despite some evidence that the distinction between the disorders is not as clear as previously argued [Bishop, 2010; Bishop, Snowling, Thompson, & Greenhalgh, 2016], whereas ADHD is currently not viewed as barring a diagnosis of DLD. The decision to exclude a diagnosis of DLD based on a diagnosis of ASD on the basis that the latter is genetic, may seem puzzling, given the observed genetic overlap between the disorders. Several years before this revision, Bishop analyzed several models in an attempt to account for the genetic overlap between SLI and ASD while maintaining that each disorder may have a distinct linguistic profile [Bishop, 2010]. The conclusion of Bishop's analysis was that a model that included genetic interaction effects could better explain the observed comorbidity of ASD and language impairment and the observed levels of language impairment in ASD and SLI probands and relatives of probands, while still incorporating potential genetic overlaps. This is in contrast to a model with purely additive genetic effects shared by both disorders (referred to as additive pleiotropic effects in the paper). In other words, Bishop's analysis showed that observational data fit a model better in which the genetic overlap between SLI and ASD was not purely additive (i.e., it is not likely the case that a genetic variant with a given additive effect on SLI risk has the same effect on ASD risk through the same mechanism).

It is interesting to note that, while a GWAS of SLI did not find genome‐wide significant associations in the analysis of the child genetic effects (as opposed to parent‐of‐origin effects), some of the top hits were in genes implicated in ASD, ADHD, or other neurodevelopmental disorders [supplementary material in Nudel, Simpson, Baird, O'Hare, Conti‐Ramsden, Bolton, Hennessy, Ring, et al., 2014a], for example, *CNTN5* [Lionel et al., 2011; Zuko et al., 2013] and *RBFOX3* [Weyn‐Vanhentenryck et al., 2014]. Given this and previous results as well as the ongoing debate as to the nature of the similarities and differences across SLI and ASD (and to a lesser degree, ADHD), we sought to use polygenic risk scores (PRSs) to explore the genetic relationship between these disorders. PRS analyses are commonly used in psychiatric genetics; in PRS analyses for ASD and ADHD, an association between PRS for ASD and cognitive ability in the general population was reported [Clarke et al., 2016]. While previous studies described incidental overlaps between SLI, ADHD, and ASD, a PRS analysis allows the examination of potential genome‐wide common variant overlaps in a systematic way. PRSs are aggregate scores capturing the additive effects of many genetic variants simultaneously. Whereas a GWAS tests one variant at a time for association with a disease or a trait, PRSs assess an individual's overall genetic risk of/predisposition to a disease or a trait based on information from a previous, independent genetic study. PRSs (as used in this study) were devised in an original study of schizophrenia [Purcell et al., 2009]. In that study, the effects of many genetic variants identified in one case–control schizophrenia sample were aggregated in individuals from an independent sample based on each individual's genotype at each locus. This aggregate score significantly predicted some of the risk in the new sample (Nagelkerke's pseudo *R* ^2^ of ~3%). Moreover, it was shown that a PRS trained on a schizophrenia sample predicted some of the risk of bipolar disorder in two independent samples (Nagelkerke's pseudo *R* ^2^ of 1.4–2%) but did not predict the risk of nonpsychiatric diseases (Nagelkerke's pseudo *R* ^2^ of ~0%). This illustrates the fact that PRSs trained on one phenotype could be used to assess the genetic relationship with another phenotype. We adopt this approach and use a GWAS of SLI as a training set to examine the genetic relationship between language impairment, SLI, ASD, and ADHD in an independent sample of children who were assessed with several cognitive test batteries, interviews, and questionnaires encompassing both behavioral and linguistic functions.

## Methods

### *Discovery GWAS*

The summary statistics used to construct the PRSs in this study are from a family based genetic study of SLI in a British cohort of SLI families [Nudel, Simpson, Baird, O'Hare, Conti‐Ramsden, Bolton, Hennessy, Ring, et al., 2014a], with a slightly updated pedigree, which included a child properly assigned as a case and a connection between two nuclear families and which was used in another previously published study [Howey et al., 2015]. The family subsets used in the discovery GWAS included 154 case‐parent trios, 53 case‐mother duos, 12 case‐father duos, and 18 cases (the numbers refer to the average counts per single‐nucleotide polymorphism (SNP)). Briefly, for comparison with the target data set, the quality control (QC) measures and phenotype criteria employed in the original study were as follows (previous reports may contain minor errors regarding some thresholds): ≥95% genotyping rate for SNPs and individuals, and ≥1% minor allele frequency (MAF) for SNPs. SNPs with a Gentrain score <0.5 were removed. Individuals and SNPs with >1% Mendelian errors were removed. Individuals with extreme heterozygosity rates (≥±3 *SD* from the mean), individuals of divergent ancestry as identified in a principal component analysis (PCA), and individuals with X or Y aneuploidies or discordant sex information were removed. The Hardy–Weinberg equilibrium (HWE) *P*‐value exclusion threshold in the final pedigree (after sample exclusion) was ≤1 × 10^−3^, as used with PLINK v1.07 [Purcell et al., 2007] taking into account only founders and disregarding affection status (as parents were not assessed for SLI), and SNPs that showed some association with SLI were checked with PEDSTATS v0.6.10 [Wigginton & Abecasis, 2005], whereupon they were removed if their HWE *P*‐value from PEDSTATS was ≤1 × 10^−3^. The GWAS was a family‐based GWAS in which only cases, case‐parent duos, and case‐parents trios were used. The SLI phenotype in the discovery GWAS was determined in the following manner: cases were (a) children who had low expressive and/or receptive language scores from the Clinical Evaluation of Language Fundamentals, revised (CELF‐R) [Semel, Wiig, & Secord, 1992]: 1.5 *SD* (or more) below the population mean for their age (i.e., a score ≤77.5) and/or (b) children who were SLI probands based on clinical evaluation (or clinically language‐impaired siblings, where the first identified child was not included). Cases had normal measures of nonverbal intelligence and no known etiology for their language impairment. It should be mentioned that an estimated one third of the children showed some evidence of ADHD or developmental coordination disorder [Newbury et al., 2009]. Further information about the cohort is available in previous reports [Falcaro et al., 2008; Newbury et al., 2009; Nudel, Simpson, Baird, O'Hare, Conti‐Ramsden, Bolton, Hennessy, Ring, et al., 2014a; The SLI Consortium, 2002, 2004]. Marker positions in hg18 from the summary statistics were converted to hg19 using a newer version of the Illumina manifest file for the same array (Illumina Human OmniExpress), cross‐referenced for marker names. Marker names were then matched by chromosome and position in hg19 with marker names in the target data set, where possible.

During the production stage, we discovered that there were markers with duplicate positions in the target dataset, and we also learned that seven individuals (one trio, and four parents) who were flagged in the PCA (the trio) or heterozygosity rate checks (the four parents) had been kept in the dataset by mistake. While our post hoc checks showed that said trio clustered well with Europeans, the four parents did seem to have extreme heterozygosity rates (although the Pi‐hat values in the IBD check for them and their children, who were not outliers, were all within the expected range). We did not have the original files used to QC the discovery sample. We therefore repeated the analyses either with all seven individuals removed (Correction 1), or with just the four parents removed (Correction 2), in both cases using the original marker IDs without converting them between datasets, and with MAF of at least 1% in the target dataset as an added QC step. The removal of samples reduced the power of the discovery GWAS, but the conclusions of the study did not change. Also, as a further sanity check for the effect of excluding the individuals, we repeated the analysis outlined in the original 2014 paper by Nudel et al., which found a genome‐wide significant hit with paternal parent of origin effects, after updating the pedigree file – this resulted in an association with effect size 0.255 or 0.256 for A (*P* = 2.918 × 10^−8^ or *P* = 3.048 × 10^−8^, for rs4280164, the top SNP, for Corrections 1 and 2, respectively), which was very similar to the reported one. However, to clear any doubt, we contrast the results of the new PRS analyses with the original ones throughout the paper. Figures for the new analyses are available in the Supplementary Figures file. [Correction added on 14 Nov 2019 after first online publication: The preceding sentences, from “During the production stage,…” through “… are available in the Supplementary Figures file” have been added.]

### *Target Sample*

The target sample was part of the Danish High Risk and Resilience Study—VIA7 [Thorup et al., 2015]. The children were selected either for having a parent with schizophrenia or bipolar disorder or as age‐matched controls (7 years of age). Pertinent to this study, the children were administered a language test, the Danish version of the Test for Reception of Grammar, TROG‐2 (Bishop, 2003b). Scores for the number of correct blocks were age‐standardized according to the norms from the Danish manual. Additionally, children underwent screening with the Danish version of the Schedule for Affective Disorders and Schizophrenia for School‐Age Children (K‐SADS) [Kaufman et al., 1997] including supplementary sections for ASD and ADHD, when indicated through the screening, based on children's scores [Ellersgaard et al., 2018]. Two phenotypes for language impairment were used in this study: a broad one, based only on the TROG‐2 score, and a narrow one, based on TROG‐2 score and employing the following exclusion criteria: a diagnosis of ASD (based on probably or definite indication from the K‐SADS as per below) and low nonverbal intelligence from the Reynolds Intellectual Screening Test (RIST) [Reynolds & Kamphaus, 2003], as per their score on the odd‐item out subtest, adjusted using Danish norms. The broad phenotype definition resulted in more cases, but they could be more heterogeneous, and the narrow phenotype definition resulted in fewer but more robustly selected cases, who were more comparable to SLI cases in previous reports.

The project was approved by the Danish Data Protection Agency and follows all laws concerning the processing of personal data. Permission to draw data from registers was granted by the Danish Ministry of Health. The study protocol was sent to the Danish Committee on Health Research Ethics, who decided that ethical approval was not needed due to the observational nature of the study. The genetic part of the study obtained ethical approval from the outset of the study and The Danish High Risk and Resilience Study—*VIA* 7 was later incorporated into the protocol (Arv og Miljø—genetics and environment) as an appendix, which has then been approved by the committee (ARV OG MILJØ: betydning for psykisk sygdom hos børn og unge (H‐B‐2009‐026)). Written informed consent was obtained from all adult participants and from the legal guardians of participating children.

### *Phenotypes in the Target Sample*

Broad language phenotype: language impairment cases were defined as having a low standardized score on the TROG‐2 test, at most 77.5 (which constitutes 1.5 *SD* below the mean, where the mean was 100 and the *SD* was 15, similar to the expressive and receptive language scores from the CELF‐R). Language impairment controls were defined as having a standardized score of at least 92.5 (which constitutes 0.5 *SD* below the mean). Individuals with scores not within those ranges were excluded from the primary PRS analyses for the language phenotypes (see Results section for more information regarding their exclusion).

Narrow language phenotype (SLI): SLI cases and controls were defined as per the above criteria, but language impairment cases whose nonverbal intelligence from the RIST was below 35 (corresponding to a threshold of 1.5 *SD* below the age‐standardized mean from the Danish norms) and language impairment cases who had an ASD diagnosis from the K‐SADS were excluded. These criteria are closer to the original SLI criteria used in the SLI Consortium studies, including the discovery GWAS.

ASD and ADHD: indications for ASD and ADHD are based on the K‐SADS lifetime criteria (i.e., current or prior to interview), which are based on the DSM‐IV (for ASD this includes a question pertaining to pragmatic language problems). Children who had a probable or definite diagnosis of ASD or ADHD were defined as ASD or ADHD cases, respectively, and children who did not have a probable or definite diagnosis of ASD or ADHD were defined as ASD or ADHD controls, respectively. All diagnostic assessments of children who were suspected of having ASD or ADHD were reviewed by a specialist in child and adolescent psychiatry.

Height: as a negative control for the prediction ability of PRS trained on SLI, we used the children's height measurements taken around age 7. Height is one of the longest‐studied traits in genetic research, and it is known to have a strong genetic component [McEvoy & Visscher, 2009]. Moreover, a large study of 45 twin cohorts that examined the genetic and environmental factors influencing height from infancy to early adulthood estimated the additive heritability of height at age 7 to be ~60% [Jelenkovic et al., 2016]. Thus, height is a good candidate for a genetic trait, and one that is not expected to be predicted by PRS trained on SLI. Since children were not all exactly the same age when measured, a covariate for the age of the child at measurement was used in the linear regression model. The covariate is used by the program in the estimation of the coefficient of the PRS, but the reported *R* ^2^ is for the effect of the PRS alone.

### *Genetic Data and Quality Control in the Target Data Set*

DNA was collected from blood or saliva samples. Samples were genotyped on the Illumina PsychChip v1.1. Following genotyping, preliminary QC of the raw data included the following steps: SNPs with a Gentrain score <0.3 were removed, as were samples with low call rates (<95%) or discordant sex information (plots were examined manually using X chromosome SNP data). Following this initial QC, PLINK pedigree files were generated and subject to QC based on the general guidelines described in Anderson et al. [2010], with some steps added or adjusted for our family‐based sample. SNPs and individuals with >1% Mendelian errors were removed. SNPs and individuals with >5% missing data were removed. Individuals with extreme heterozygosity rates (≥±3 *SD* from the mean) were removed. A relationship matrix was computed with PLINK v1.9b5.2. The Pi‐hat threshold for the exclusion of unrelated individuals was 0.185. Families with individuals showing cryptic relatedness or with relatives who had unexpectedly low Pi‐hat values were removed. A PCA was performed with PLINK using genetic data from the HAPMAP populations included in the QC protocol [Anderson et al., 2010]. Following this, the threshold for the exclusion of samples was 2 *SD* above or below the VIA7 mean for either PC1 or PC2. Relatives of individuals of divergent ancestry were also removed. Lastly, a HWE *P*‐value exclusion threshold of 1 × 10^−6^ was employed for QC‐passing SNPs taking into account only founders and disregarding affection status. Given the type of array used to genotype the VIA7 samples, which also included rare variants, no filtering based on MAF was performed at this stage. Following QC, 1,094 individuals from 429 families remained.

### *Polygenic Risk Scores*

**Figure 1:**
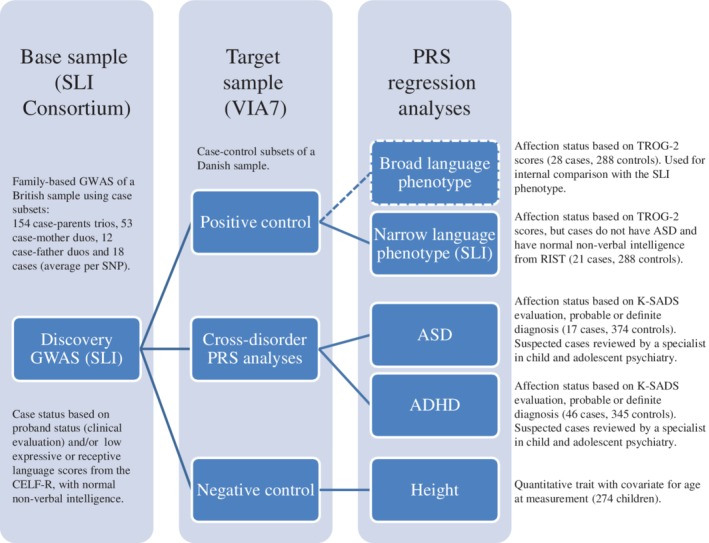
An outline of the study design. CELF‐R: Clinical Evaluation of Language Fundamentals, revised; PRS, polygenic risk score; TROG‐2, test for reception of grammar‐2; RIST, Reynolds intellectual screening test; K‐SADS, schedule for affective disorders and schizophrenia for school‐age children.

The discovery GWAS was re‐run with a newer version of EMIM (v3.22) [Howey & Cordell, 2012] and the updated pedigree file. For every SNP from the GWAS, EMIM outputs *R*_1_, the increase in the risk an individual has above baseline risk, if he or she carries one risk allele (the minor allele is by default the “risk,” or effect, allele, and, therefore, *R*_1_ can also be smaller than 1, if the minor allele is protective). In the GWAS model which was run, it was assumed that the increase in the risk when carrying two risk alleles was *R*_1_^2^, so only one risk parameter was estimated in the full model. *R*_1_ was used as the effect size (similar to OR) in the construction of the PRS in the target data set. EMIM also outputs a warning for SNPs, if some problem arose during the likelihood estimation for that SNP. All SNPs for which such a warning was obtained were removed from the base data set, as were A/T and G/C SNPs. Further QC steps for the target data set of SNPs, as well as the calculation of the PRS and the regression of the phenotypes on them, were performed with PRSice v2.2.3 [Choi & O'Reilly, 2019]. Mismatched SNPs were removed by PRSice, and SNPs were then clumped using an *r* ^2^ threshold of 0.2 in a window of 500 kbp, as recommended for studies of psychiatric phenotypes [Wray et al., 2014]. Additionally, SNPs in the MHC region (chromosome 6, 28,477,797‐33,448,354, hg19) were removed from the target data set due to the complex linkage disequilibrium pattern in the MHC region. As our target sample was small, we were underpowered to test several *P*‐value thresholds. Therefore, all QC‐passing SNPs from the discovery GWAS were considered before downstream procedures by PRSice (i.e., the *P*‐value threshold was 1). This approach was chosen because, in small samples, the inclusion of all SNPs maximizes the predictive ability of the PRS, because including all SNPs makes no assumption about the distribution of effects [Dudbridge, 2013], and, lastly, because, for highly polygenic traits, the inclusion of all SNPs typically results in better prediction of trait variance, as was shown in an evaluation of several PRS protocols across several traits [Ware et al., 2017]. PRSice reports Nagelkerke's pseudo *R* ^2^ (*R* ^2^_N_) as a measure of the goodness of fit of the model. Additionally, prevalence values of 10% for the broad language phenotype [Norbury et al., 2016], 7% for the narrow language phenotype (SLI) [Tomblin et al., 1997], 5% for ADHD [Polanczyk, de Lima, Horta, Biederman, & Rohde, 2007], and 1% for ASD [Baird et al., 2006] were used by PRSice to adjust the *R* ^2^ value for the prevalence and the proportion of cases in the sample, thus also accounting for a potential bias in the ascertainment of cases, even though the sample was not ascertained for language impairment, ASD or ADHD *per se*, and, at the same time, transforming the *R* ^2^ to the liability scale [Lee, Goddard, Wray, & Visscher, 2012]. As the discovery study used both male and female children with SLI (and the association model did not allow the inclusion of a covariate for sex), children of both sexes were used in the PRS analyses. An outline of the study design, including brief descriptions of all sample subsets, is shown in Figure 1.

## Results

The discovery GWAS that used the slightly updated pedigree did not obtain very different results from the original one; no genome‐wide significant associations were identified. The top hits were still in genes implicated in other neurodevelopmental disorders (Supplementary Table S1).

**Figure 2:**
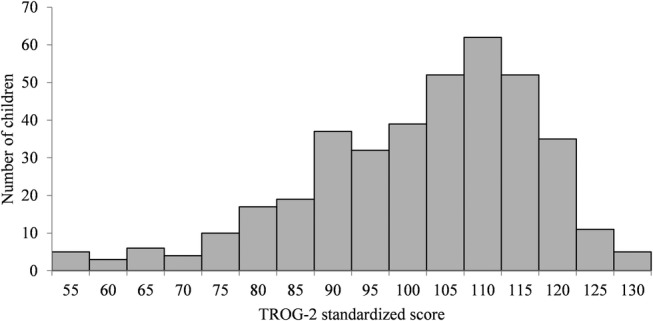
TROG‐2 test scores in the QCed genotyped sample. TROG‐2, test for reception of grammar‐2.

After excluding parents and siblings from the QCed target data set, the total sample included 391 unrelated children, with the oldest child having been born in March 2005, and the youngest in January 2009 (note: the tests were administered several years before this study was performed). The distribution of the language test scores in the sample is shown in Figure 2. The sample mean was 101.17, with *SD* = 15.69. The proportions of cases in each group were 7.1%, 5.4%, 4.3%, and 11.8%, for the broad language phenotype, the narrow language phenotype (SLI), ASD, and ADHD, respectively. However, the proportions of cases excluding children with an “unknown” phenotype (those who have not taken the test or who fell outside the range for being a case or a control for the language phenotypes) were 8.9% and 6.8% for the two language phenotypes, respectively. These values are close to the reported prevalence for SLI, but they are slightly higher than the population prevalence for ASD and ADHD. This may be because some of the children in the study were selected for a family history of psychiatric disorders, which are hypothesized to be closely related to ASD and ADHD [Matson & Nebel‐Schwalm, 2007; Owen, O'Donovan, Thapar, & Craddock, 2011] and/or because “probable cases” were also included.

**Figure 3:**
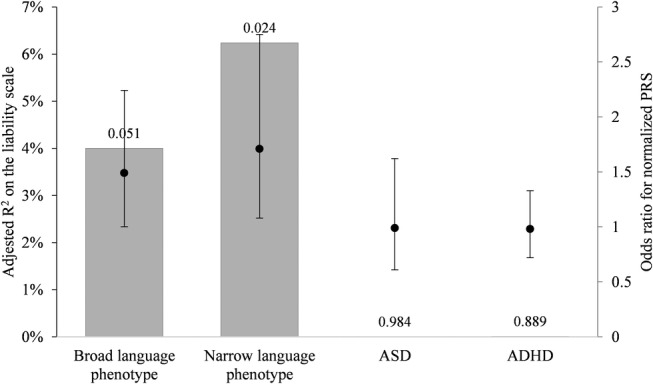
Results of the PRS analyses for the language phenotypes, ASD and ADHD; the gray columns represent the prevalence‐adjusted *R* ^2^ on the liability scale and the *P*‐values for the regression coefficient of the PRS are given above each column. The black dots represent the odds ratios for the normalized PRS from the logistic regression, with 95% confidence intervals calculated using the confint function in R. ASD, autism spectrum disorder; ADHD, attention deficit/hyperactivity disorder; PRS, polygenic risk score.

Following the QC and clumping procedures, the number of SNPs used in the construction of the PRS was 84,270 (Correction 1: 80,167 SNPs; Correction 2: 80,130 SNPs). [Correction added on 14 Nov 2019 after first online publication: in the preceding sentence, the text in the parentheses has been added.]. The results show that the PRS trained on the SLI sample predicts some risk of the broad language phenotype and significantly predicts some of the risk of the narrow language phenotype (SLI), with adjusted *R* ^2^ = 4% and 6.24%, *P* = 0.051 and 0.024, respectively (in both cases the association with PRS was positive i.e. the regression coefficient for PRS was positive). However, it does not predict risk of ASD or ADHD (adjusted *R* ^2^ = 0.0004%, 0.01%; *P* = 0.984, 0.889, respectively), as can be seen in Figure 3 and Table 1, which also shows the different sample sizes. The results for the corrected analyses showed similar trends, see Table 1 for details. [Correction added on 14 Nov 2019 after first online publication: the preceding sentence was added.] The PRS analysis with height as the target phenotype included 274 children with confirmed non‐missing height phenotype and covariate for age at measurement and resulted in an *R* ^2^ of 0.2% (*P* = 0.452). The results for the new analyses for height were: *R* ^2^ of 0.019%, 0.061%; *P* = 0.814, 0.676, for Corrections 1 and 2, respectively. [Correction added on 14 Nov 2019 after first online publication: the preceding sentence was added.] The above two‐sided *P*‐values are based on the degree to which the PRS regression coefficients are different from zero, estimated using a Wald test (a *t*‐test in the case of height, as it is estimated in a linear regression; otherwise, the normal distribution is used to obtain the *P*‐value for the coefficient from a logistic regression). Figure 3 also shows the odds ratios from the logistic regression coefficients and their confidence intervals, computed in R v3.4.2 [R Core Team, 2014] using PRS normalized across all children for the four neurodevelopmental phenotypes. The TROG‐2 thresholds chosen for defining cases and controls conform to thresholds used in previous SLI Consortium studies and to the qualitative assessment in the Danish version of the TROG‐2 manual, where scores in the 85–90 range are considered “lower part of the average.” However, we recognize that excluding children whose scores were higher than 77.5 but lower than 92.5 could bias the results of the regressions for the two language phenotypes and could also result in a loss of power. We also note, however, that the discovery GWAS did not use controls, but, rather, family‐based case subsets, which means this issue would not have affected the weights used in the construction of the PRS. That said, we examined how defining the above group of children as controls, instead of excluding them, might affect the results: for the narrow language phenotype (SLI), a Nagelkerke's pseudo *R* ^2^ of 4.92% (*P* = 0.012) was obtained (3.95%, 4.18%; *P* = 0.023, 0.02, for Corrections 1 and 2, respectively); for the broad language phenotype, a Nagelkerke's pseudo *R* ^2^ of 3.29% (*P* = 0.024) was obtained (2.69%, 2.73%; *P* = 0.04, 0.039, for Corrections 1 and 2, respectively). [Correction added on 14 Nov 2019 after first online publication: In the preceding sentence, the text in parentheses following “… (*P* = 0.012) was obtained” and the text in parentheses following “… (*P* = 0.024 was obtained” was added. The sentence “In the previous analyses, these were 4.34% and 2.73%, respectively (Table 1),” which followed the preceding sentence, was deleted.] We also examined how adding a covariate for whether the child is from a VIA7 high risk family (i.e., a family in which at least one parent had a diagnosis of schizophrenia or bipolar disorder) might affect the results (we note that at least 75% of the children in each of the case groups come from a high risk family); while adding a covariate resulted in a higher Nagelkerke's pseudo *R* ^2^ for the models of all neurodevelopmental phenotypes, indicating that the covariate for high risk status explained some of the risk and thus improved the models, the only model in which the PRS was significantly associated with the outcome was the one for the narrow language phenotype (SLI) (*P* = 0.043); this did not pass the 0.05 threshold for Corrections 1 and 2 (*P* = 0.068, 0.059, respectively), due to reduced power. [Correction added on 14 Nov 2019 after first online publication: In the preceding sentence, the text “just as before” was deleted following “(*P* = 0.043)”, and the text that follows “(P = 0.043)” was added.].

**Table 1:** Results of the PRS Analysis for Binary Traits

Trait	*N* (cases)	*R* ^2^_N_ (%)	Adjusted *R* ^2^ on the liability scale (%)	*P*
Broad language phenotype	316 (28)	2.73/2.33/2.4	4/3.41/3.51	0.051/0.07/0.066
Narrow language phenotype (SLI)	309 (21)	4.34/3.59/3.86	6.24/5.21/5.61	0.024/0.038/0.032
ASD	391 (17)	0.0004/0.015/0.003	0.0004/0.015/0.003	0.984/0.893/0.955
ADHD	391 (46)	0.01/0.009/0.045	0.01/0.009/0.048	0.889/0.895/0.763

R^2^_N_, Nagelkerke's pseudo R^2^.

The results are given for the original analysis/ Correction 1/ Correction 2. [Correction added on 14 Nov 2019 after first online publication: All values under the last three columns of the table have been updated.].

**Figure 4:**
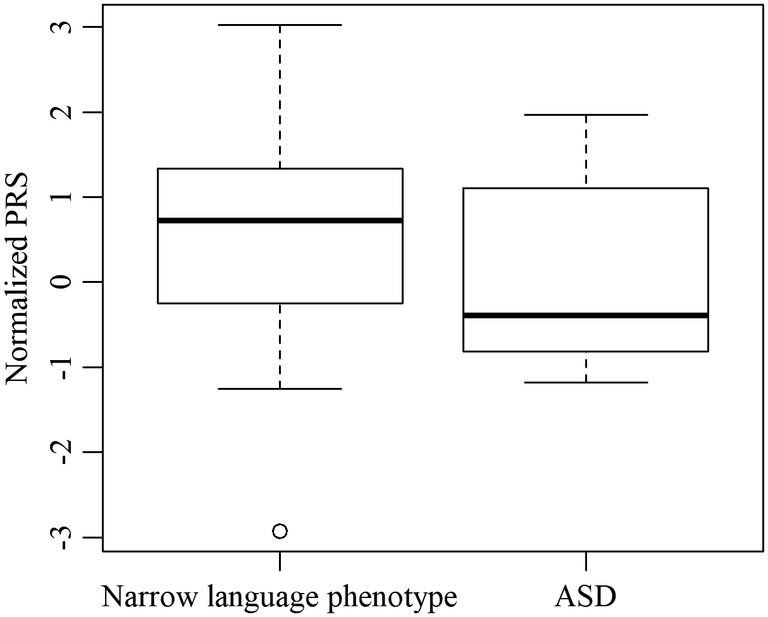
Boxplot of normalized PRS in children with the narrow language phenotype and children with ASD. ASD, autism spectrum disorder; PRS, polygenic risk score.

The narrow language phenotype (SLI) and ASD case groups were mutually exclusive; to test whether they significantly differed with respect to their PRSs, we employed a Wilcoxon rank sum test (Mann–Whitney *U* test) in R using the normalized PRS. As per the way the PRSs are constructed, if they are indeed predictive, then individuals with the trait in question should have higher PRSs than individuals without it. We therefore tested whether the PRSs of children with the narrow language phenotype (SLI) are shifted to the right (i.e., are higher) compared to the PRSs of children with ASD. We found that this was indeed the case (*W* = 237, one‐sided *P* = 0.044, common language effect size = 0.66, difference in location = 0.667, 95% lower confidence bound = 0.026). Given the small sample size for this analysis and the reduced power of the GWAS for Corrections 1 and 2, the differences were not significant when using PRS from those analyses, but they were of comparable size: difference in location of 0.62, 0.54 (*P* = 0.07, 0.053), respectively. [Correction added on 14 Nov 2019 after first online publication: The preceding sentence was added.] Boxplots for the normalized PRS in both groups are shown in Figure 4. Figures for Corrections 1 and 2 are available in the Supplementary Figures file. [Correction added on 14 Nov 2019 after first online publication: The preceding sentence was added.]

## Discussion

In this study, we utilized a sample of Danish children tested for linguistic and behavioral traits to examine the genetic relationship between SLI, ASD, and ADHD; SLI involves a primary deficit in language development, whereas in ASD and ADHD language and communication may be impaired as well, but they are characterized with broader behavioral phenotypes.

To what degree do neurodevelopmental disorders in which language may be affected relate to each other? This question can be addressed in different ways. Previous studies have, for the most part, focused on the linguistic angle. Genetic studies have focused mostly on specific genes implicated in more than one disorder. Here, we attempted to shed more light on this issue using a genome‐wide approach. Our results show that PRSs trained on a GWAS of SLI significantly predicted some risk of language impairment in an independent sample but did not do so for risk of ASD or ADHD. Additionally, we observed a significant difference between the normalized PRS in children with the narrow language phenotype (SLI) and the normalized PRS in children with ASD. Overall, this could suggest that genetic overlaps across these disorders (at least those based on common variants) are not purely additive by nature, that is, variants that additively increase the risk of SLI do not necessarily increase the risk of ASD in the same fashion (even though the ASD diagnosis may have included some form of language problem, likely of a pragmatic nature or delayed development). This does not mean that all genetic overlaps between SLI, ASD, and ADHD are of this nature; indeed, even the discovery GWAS on which this PRS study was based had obtained top hits in genes implicated in ASD and ADHD, as did other studies, as mentioned earlier. However, these genes were at times implicated in different ways, for example, SNP‐based analyses, copy number‐based analyses, rare variant‐based analyses, and so on. This makes it difficult to assess whether the underlying mechanism driving the association is the same across all disorders. Moreover, it has been suggested that several distinct PRS procedures could represent potentially different genetic explanations, as they sometimes appear uncorrelated in the same data set [Ware et al., 2017]. The top hits in the discovery GWAS, or other reported associations, might still have some additive effects across SLI and ASD or ADHD, but our results suggest that the overall effect of many “SLI loci” together on ASD and ADHD risk is not purely additive, or, at least, that their effects are not the same across SLI and ASD and ADHD. It should also be noted that the autism spectrum is heterogeneous and includes both children with severely affected language (e.g., children diagnosed with childhood autism) and children with relatively high language skills (e.g., children diagnosed with Asperger syndrome). Nonetheless, studies have indicated that even children with Asperger syndrome may have deficits in receptive language [Koning & Magill‐Evans, 2001; Saalasti et al., 2008]. It would be interesting to examine different subtypes of ASD separately given adequate samples are available.

Our study may also be relevant to the question of what criteria should be used in genetic research of language impairment. The larger adjusted *R* ^2^ observed when using stricter criteria to define the language phenotype (which are closer to the original SLI criteria) could imply that, at least, when it comes to genetic research, there is some value in continuing to use more specific phenotypes (this is not to say the difference between the estimates was significant in this study). That having been said, it should be noted that the broad language phenotype here is not fully congruent with the definition of DLD, which would require a more clinical assessment of the child's linguistic profile.

One of the strengths of this study is the thoroughly phenotyped, linguistically and genetically homogenous sample (in terms of ancestry) used as the target data set. It is not common to have one study sample assessed for language traits, ASD and ADHD, where all individuals have also undergone genotyping together. This allowed us to perform comparisons across disorders while minimizing differences that may otherwise occur when using different samples, different genotyping platforms, different diagnostic measures, and so on. Despite this, it should be noted that the target sample was not ascertained for language impairment, in contrast to the sample used in the discovery GWAS, nor was it ascertained for ASD or ADHD. We did not have clinical data on language ability or an expressive language score, both of which were used, in addition to a receptive language score, in defining SLI cases in the discovery GWAS. This could imply that we potentially had some children defined as controls, when they could have been defined as cases had those measures been available. Another consequence of the above is that we had a small number of language impairment, ASD, and ADHD cases, which could be viewed as the major limitation of this study. Especially for SLI, genotyped samples are very scarce, with the largest sample (to the best of our knowledge) being the one used in the discovery GWAS. That said, it should be noted that large enough effect sizes in some samples but not others (of similar sizes) across the phenotypic groups were observed, and, additionally, there was a significant difference in the PRS between groups (for SLI and ASD). Lastly, it should be noted that the VIA7 families were ascertained for parental status of schizophrenia or bipolar disorder (or as control families of age‐matched children). While the sample was not ascertained for the phenotypes studied here, we do observe slightly higher rates of ASD and ADHD in the sample compared to the general population. As discussed earlier, this could be the result of some connection between these disorders and the psychiatric history of the parents or the diagnostic criteria, but we note again that most of the children in each of the case groups come from families where at least one parent was diagnosed with schizophrenia or bipolar disorder. Since this was observed across all case groups, and the parent's having a diagnosis does not mean the child would necessarily receive one in the future, we do not believe that the differences in the PRS analyses across the different groups can be fully explained by this observation. [Correction added on 14 Nov 2019 after first online publication: The text “we also repeated the regression analyses while adding a covariate for this, and the only model in which the PRS had a coefficient significantly different from zero was still the one for the narrow language phenotype (SLI)” was deleted from the end of the preceding sentence.] However, it would nonetheless be useful to examine the genetic overlap between language disorders and psychiatric disorders, given suitable samples are found, particularly in light of some studies having reported social difficulties in children with SLI [Conti‐Ramsden & Botting, 2004] as well as an increased risk of psychiatric disorder in adulthood in individuals diagnosed with language disorders in in childhood [Clegg, Hollis, Mawhood, & Rutter, 2005]. While no studies looked at genetic correlations between SLI and psychiatric disorders, ASD, ADHD, schizophrenia, and bipolar disorder were included in a study examining genetic correlations across disorders; the latter two were highly correlated, but there was also a low but significant genetic correlation between ASD and schizophrenia [Lee et al., 2013]. Phenotypic overlaps between the two disorders have also been observed [King & Lord, 2011].

In conclusion, we believe that this study could offer a new insight into the underlying molecular mechanisms of the language deficits across these three neurodevelopmental disorders. Our results suggest that common genetic variants influencing SLI risk do not primarily contribute to ASD or ADHD in a purely additive way. This study contributes to ongoing neurogenetic research and can inform linguistic theories on the etiologies and underlying mechanisms of these conditions. Furthermore, we hope that this study will encourage further, larger studies into the genetic similarities and differences across these disorders, which could also potentially benefit clinical practice in the future.

## Conflict of Interest

The authors have no conflicting interests to declare, but T.W. states that he has acted as a lecturer and scientific counselor to H. Lundbeck A/S.

## Authors Contributions

R.N. conceived the study, performed the discovery GWAS, defined and processed the language phenotypes, performed the QC of the target data set genetic data, performed the PRS analyses, analyzed the results, wrote the manuscript; M.J.U. performed the transformation of language test scores to norm‐based scores; J.O. performed data management for VIA7 and assisted with the QC of the pedigree information; C.A.J.C., N.H., D.V.E., K.S.S., B.K.B., A.N.G., and D.L.G. contributed to the VIA7 data collection and/or pilot study; J.‐B.G. oversaw sample preparation and genotyping and performed initial QC on raw genetic data of the target data set; A.A.E.T., J.R.M.J., O.M., and M.N. contributed to the conception of the VIA7 study and its design, coordination and funding applications; T.W. designed and oversaw the genetic part of the VIA7 study.

## Supporting information


**Supplementary Table S1**: Top 10 SNPs in the discovery GWAS, comparing the original GWAS and the GWAS with the updated pedigree.

**Supplementary Figures**: Figures for the new analyses, after the exclusion of individuals from the discovery GWAS. [Correction added on 14 Nov 2019 after first online publication: The Supplementary Figures have been added.]
