# Supplementary material for: Language deficits in specific language impairment, attention deficit/hyperactivity disorder, and autism spectrum disorder: An analysis of polygenic risk
Source: Autism Res. 2019 Oct 2;13(3):369–81. doi: 10.1002/aur.2211 (PMC7078922; doi:10.1002/aur.2211)
Supplement: Supplementary file 1 — Supplementary Table S1: Top 10 SNPs in the discovery GWAS, comparing the original GWAS and the GWAS with the updated pedigree. [file AUR-13-369-s001.docx]

Table S1: Top 10 SNPs in the discovery GWAS, comparing the original GWAS and the GWAS with the updated pedigree.

| SNP | Rank in new EMIM analysis | EMIM_risk_allele | lnR1 | P | Gene | Rank in old EMIM analysis |
| --- | --- | --- | --- | --- | --- | --- |
| rs7109365 | 1 | G | 0.87413 | 1.94E-06 | CNTN5 | 2 |
| rs4790018 | 2 | G | 0.66503 | 3.05E-06 | RBFOX3 | 1 |
| rs11876129 | 3 | G | -1.19308 | 6.01E-06 |  | 3 |
| rs574715 | 4 | G | 0.63616 | 7.83E-06 |  | 4 |
| rs4266409 | 5 | A | -1.44746 | 8.99E-06 |  | 9 |
| rs671986 | 6 | G | 0.62398 | 1.15E-05 |  | 5 |
| rs9874037 | 7 | A | -1.01962 | 1.15E-05 | THRB | 11 |
| rs7760531 | 8 | G | -0.62709 | 1.42E-05 | MDGA1 | 8 |
| rs7827167 | 9 | C | -0.60725 | 2.1E-05 | TUSC3 | 6 |
| rs1076929 | 10 | A | 0.6507 | 2.17E-05 | ETV7 | 18 |
| rs4977291 | 12 | A | 1.01128 | 2.4E-05 | DENND4C | 7 |
| rs4977517 | 15 | G | 0.95965 | 3.11E-05 | DENND4C | 10 |
