# Supplementary material for: Language deficits in specific language impairment, attention deficit/hyperactivity disorder, and autism spectrum disorder: An analysis of polygenic risk
Source: Autism Res. 2019 Oct 2;13(3):369–81. doi: 10.1002/aur.2211 (PMC7078922; doi:10.1002/aur.2211)

Figures corresponding to the analyses shown in Figures 3 and 4 in the paper are included here for both sets of analyses

Excluding all seven individuals:

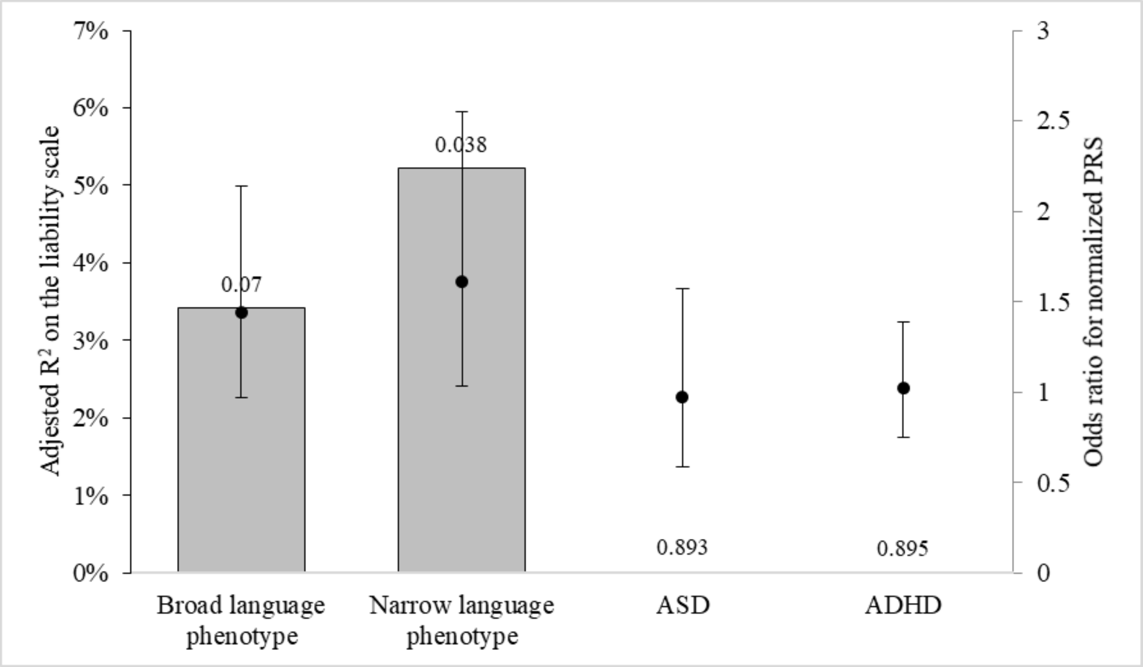

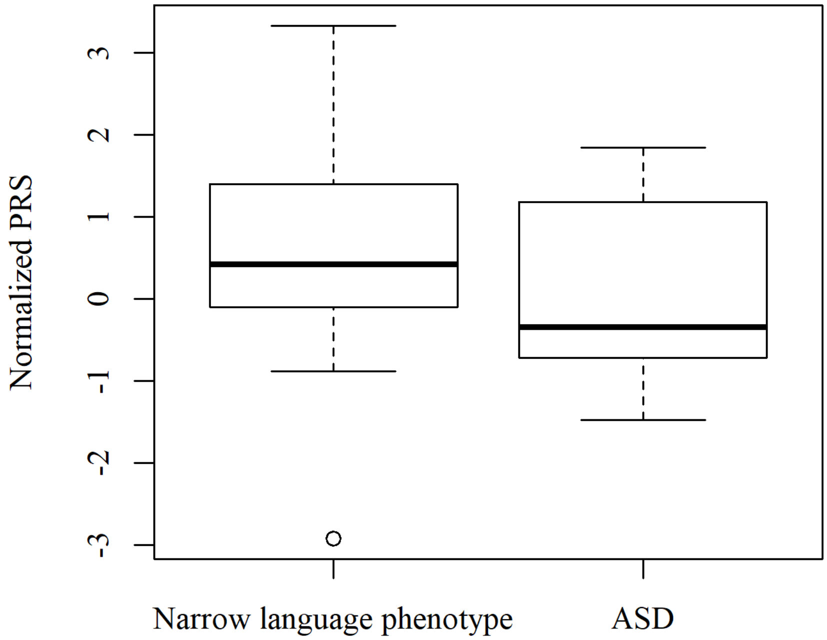

Figures corresponding to the analyses shown in Figures 3 and 4 in the paper are included here for both sets of analyses

Excluding only the four heterozygosity rate outliers:

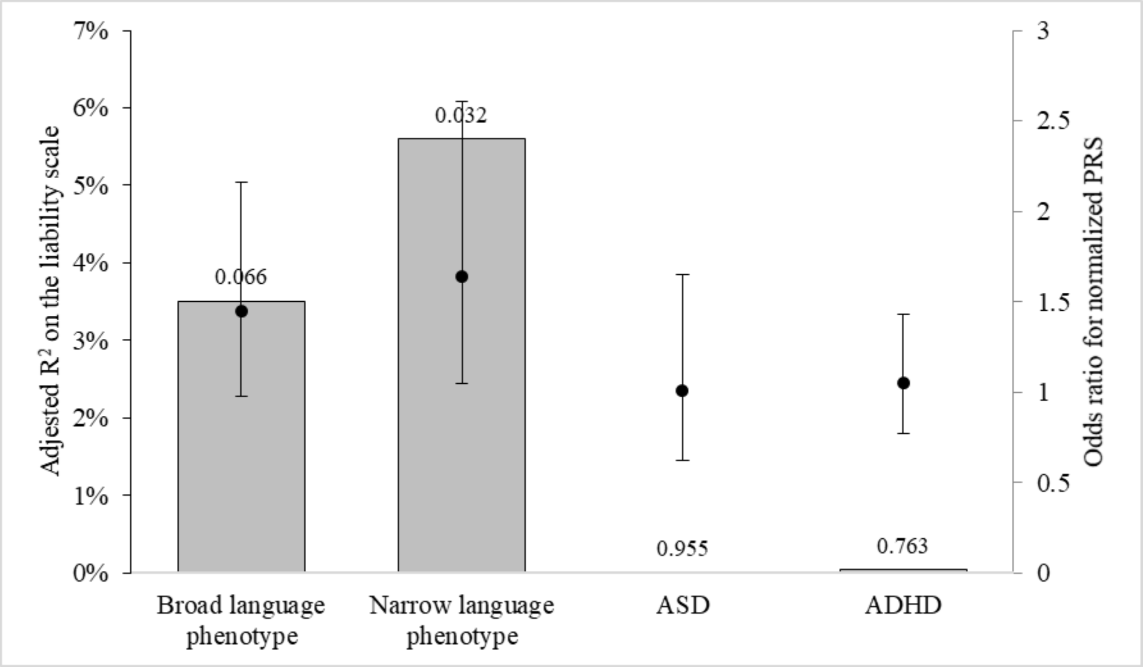

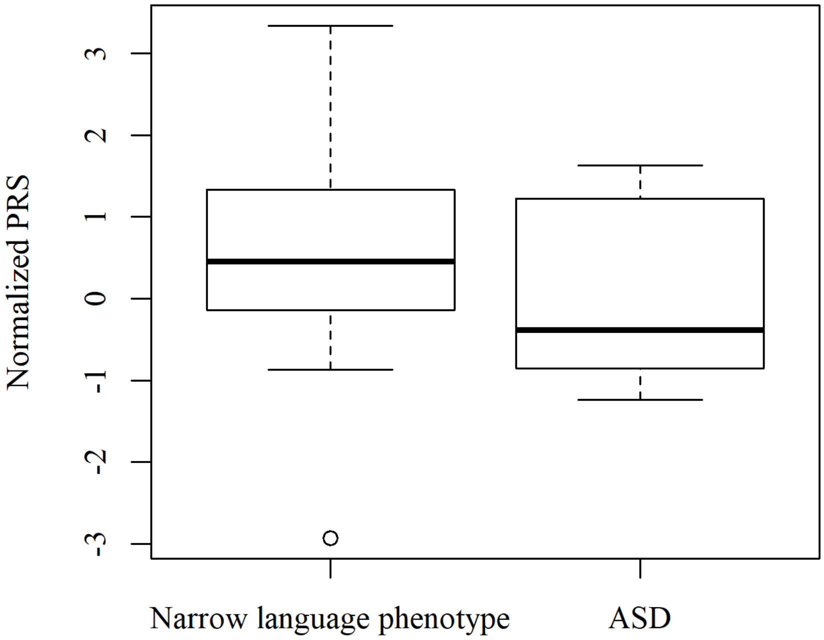

Supplement: Supplementary file 2 — Supplementary Figures: Figures for the new analyses, after the exclusion of individuals from the discovery GWAS. [Correction added on 14 Nov 2019 after first online publication: The Supplementary Figures have been added.] [file AUR-13-369-s002.pdf]
